# Supplementary material for: Effect of Different Processing Methods on the Chemical Constituents of Scrophulariae Radix as Revealed by 2D NMR-Based Metabolomics
Source: Molecules. 2022 Jul 22;27(15):4687. doi: 10.3390/molecules27154687 (PMC9331298; doi:10.3390/molecules27154687)
Supplement: Supplementary file 1 [file molecules-27-04687-s001.zip › molecules-1821627-supplementary.pdf]

Supporting Information

Effect of Different Processing Methods on the Chemical Constituents of Scrophulariae Radix as Revealed by 2D NMR-Based Metabolomics

Xiaohui Duan [1,2], Mina Zhang [1], Huan Du [3], Xiu Gu [3], Caihong Bai [3], Liuqiang Zhang [1], Kaixian Chen [1], Kaifeng Hu [2,*], Yiming Li [1,*]

[1] School of Pharmacy, Shanghai University of Traditional Chinese Medicine, 1200 Cailun Road, Shanghai 201203, China; duanxiaohui2019@163.com (X.D.); mina1215737003@163.com (M.Z.); 04100217@163.com (L.Z.); kxchen@simm.ac.cn (K.C.)

[2] Innovative Institute of Chinese Medicine and Pharmacy, Chengdu University of Traditional Chinese Medicine, 1166 Liutai Avenue, Chengdu 611137, China

[3] School of Pharmacy, Chengdu University of Traditional Chinese Medicine, Chengdu 611137, China; dave911dh@163.com (H.D.); guxiu@stu.cdutcm.edu.cn (X.G.); baicaihong@stu.cdutcm.edu.cn (C.B.)

* Correspondence: kaifenghu@cdutcm.edu.cn (K.H.); ymlius@163.com (Y.L.); Tel.: +86-28-61656141 (K.H.); Fax: +8628-87784606 (K.H.); Tel.: +86-21-51322222 (Y.L.); Fax: +86-21-51322222 (Y.L.)

Contents

Table S1 Detailed information on *Scrophularia ningpoensis* Hemsl sample processing.

Table S2 Absolute segmental integrals information of ^{1}H NMR spectra of SR extracts from five solvents.

Table S3 Performance parameters R^2(cum) and Q^2(cum) of OPLS-DA models.

Figure S1 Results of optimum ratio of sample weight to solvent volume.

Figure S2 Results of optimum amount of Scrophulariae Radix used for per NMR sample of 60% methanol: water solutions.

Figure S3 Boxplot of intensities of unidentified differential features at different steaming time.

Figure S4 The permutation plot for the PLS model.

Figure S5 Boxplot of 32 unidentified differential variables in Scrophulariae Radix processed by different methods.

Table S1. Detailed information on *Scrophularia ningpoensis* Hemsl sample processing.

No.	Processing methods	Sample code
1-3	vacuum freeze drying	FD, PPS1-3
4-6	vacuum freeze drying	FD, PDS1-3
7-9	vacuum freeze drying	FD, PFS1-3
10-12	Steaming for 1 hour	S01, PPS1-3
13-15	Steaming for 1 hour	S01, PDS1-3
16-18	Steaming for 1 hour	S01, PFS1-3
19-21	Steaming for 2 hour	S02, PPS1-3
22-24	Steaming for 2 hour	S02, PDS1-3
25-27	Steaming for 2 hour	S02, PFS1-3
28-30	Steaming for 4 hour	S04, PPS1-3
31-33	Steaming for 4 hour	S04, PDS1-3
34-36	Steaming for 4 hour	S04, PFS1-3
37-39	Steaming for 8 hour	S08, PPS1-3
40-42	Steaming for 8 hour	S08, PDS1-3
43-45	Steaming for 8 hour	S08, PFS1-3
46-48	Steaming for 12 hour	S12, PPS1-3
49-51	Steaming for 12 hour	S12, PDS1-3
52-54	Steaming for 12 hour	S12, PFS1-3
55-57	Steaming for 24 hour	S24, PPS1-3
58-60	Steaming for 24 hour	S24, PDS1-3
61-63	Steaming for 24 hour	S24, PFS1-3
64-66	Steaming for 48 hour	S48, PPS1-3
67-69	Steaming for 48 hour	S48, PDS1-3
70-72	Steaming for 48 hour	S48, PFS1-3
73-75	Steaming for 72 hour	S72, PPS1-3
76-78	Steaming for 72 hour	S72, PDS1-3
79-81	Steaming for 72 hour	S72, PFS1-3
82-84	hot-air drying	HD, PPS1-3
85-87	hot-air drying	HD, PDS1-3
88-90	hot-air drying	HD, PFS1-3
91-93	sweating	SW, PPS1-3
94-96	sweating	SW, PDS1-3
97-99	sweating	SW, PFS1-3

Table S2. Absolute segmental integrals information of ^{1}H NMR spectra of SR extracts from five solvents.

Solvents / Integration of region	10.00-5.60	5.60-3.33	3.30-0.05	sum
20% methanol: water solutions	7.32E+12	4.03E+14	3.19E+13	4.43E+14
40% methanol: water solutions	7.90E+12	4.02E+14	3.34E+13	4.44E+14
60% methanol: water solutions	8.34E+12	4.11E+14	3.52E+13	4.54E+14
80% methanol: water solutions	8.09E+12	4.04E+14	3.98E+13	4.52E+14
100% methanol: water solutions	5.64E+12	1.50E+14	3.39E+13	1.90E+14

Table S3. Performance parameters R^2(cum) and Q^2(cum) of OPLS-DA models.

OPLS-DA model	R^2(cum)	Q^2(cum)
FDS01	0.998	0.834
FDS02	0.992	0.844
FDS04	0.910	0.820
FDS08	0.957	0.861
FDS12	0.970	0.886
FDS24	0.979	0.949
FDS48	0.980	0.948
FDS72	0.962	0.958

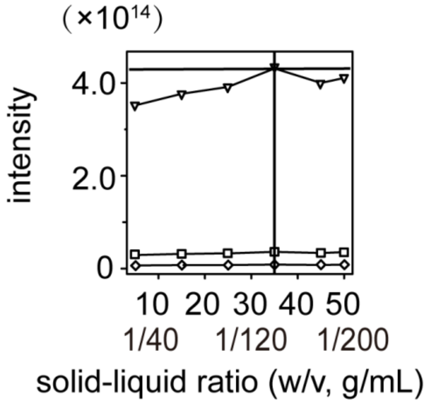

Figure S1. Results of optimum ratio of sample weight to solvent volume. Segmented integrals for aromatic region (10.00-5.60 ppm, ◇), polar (sugar) region (5.60-3.32 ppm, ▽), and aliphatic region (3.30-0.05 ppm, □) of 1D ^{1}H NMR spectra of 60% methanol: water solutions extracts of 250 mg of ground Scrophulariae Radix at different ratios of sample weight to solvent volume. The optimum extraction solvent volumes (or ratio of sample weight to solvent volume) is indicated by the vertical lines.

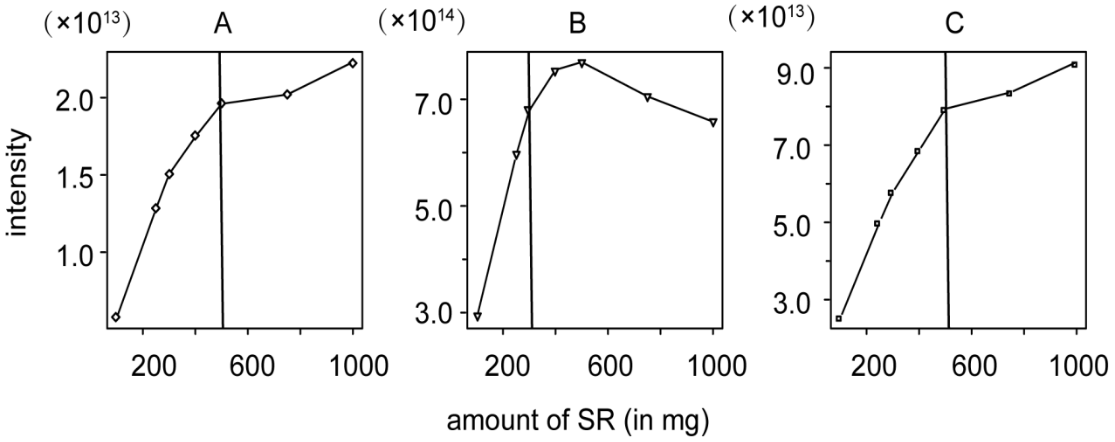

Figure S2. Results of optimum amount of Scrophulariae Radix used for per NMR sample of 60% methanol: water solutions. Segmented integrals for aromatic region (A, 10.00-5.60 ppm, ◇), polar (sugar) region (B, 5.60-3.32 ppm, ▽), and aliphatic region (C, 3.30-0.05 ppm, □) of 1D ^{1}H NMR spectra of 60% methanol water solutions of 100 mg, 250 mg, 300 mg, 400 mg, 500 mg, 750 mg, 1000 mg of Scrophulariae Radix. Optimum amounts of Scrophulariae Radix used for per NMR sample of

250 mg are indicated by the vertical lines, which are located in the linear range.

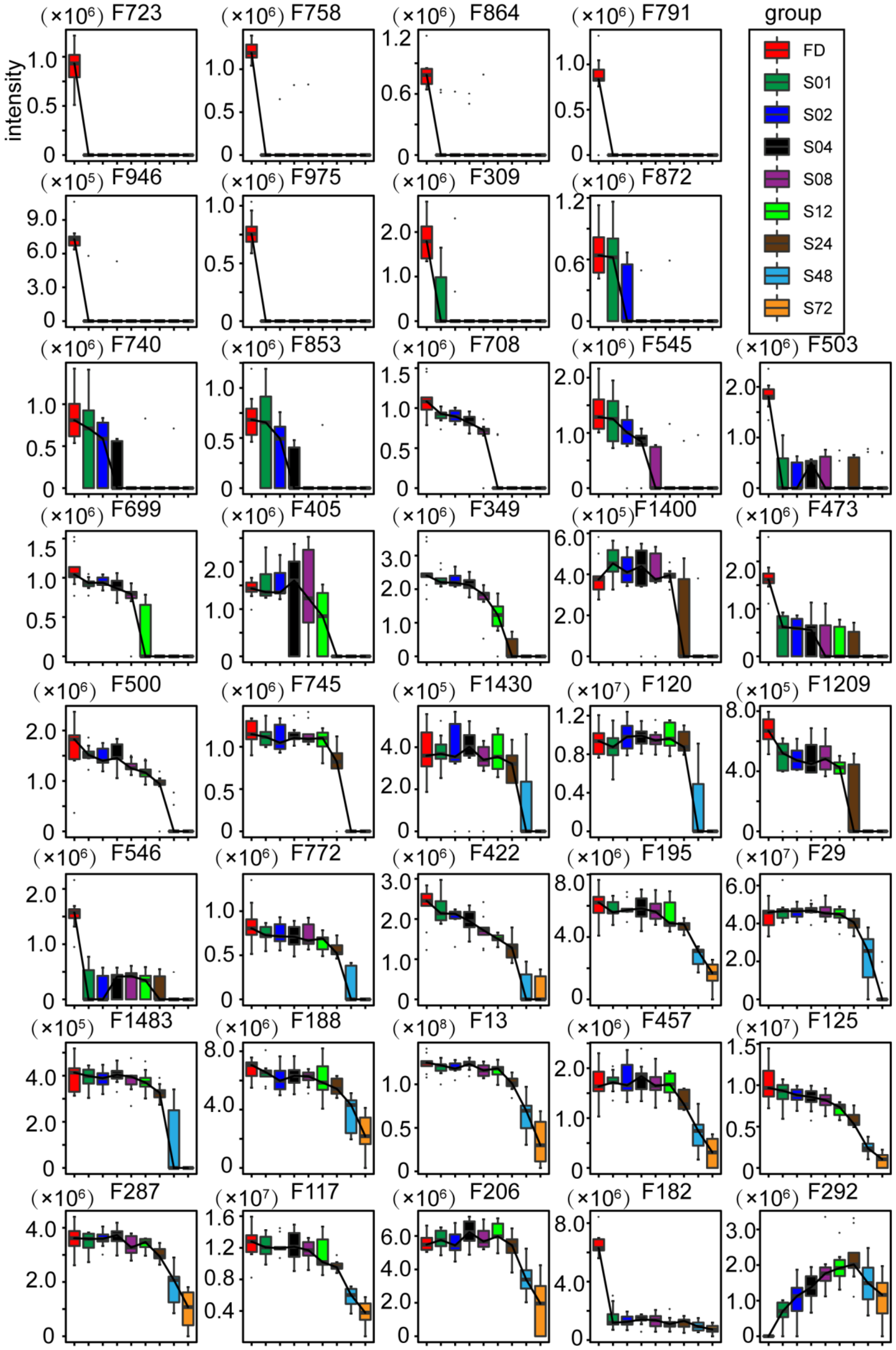

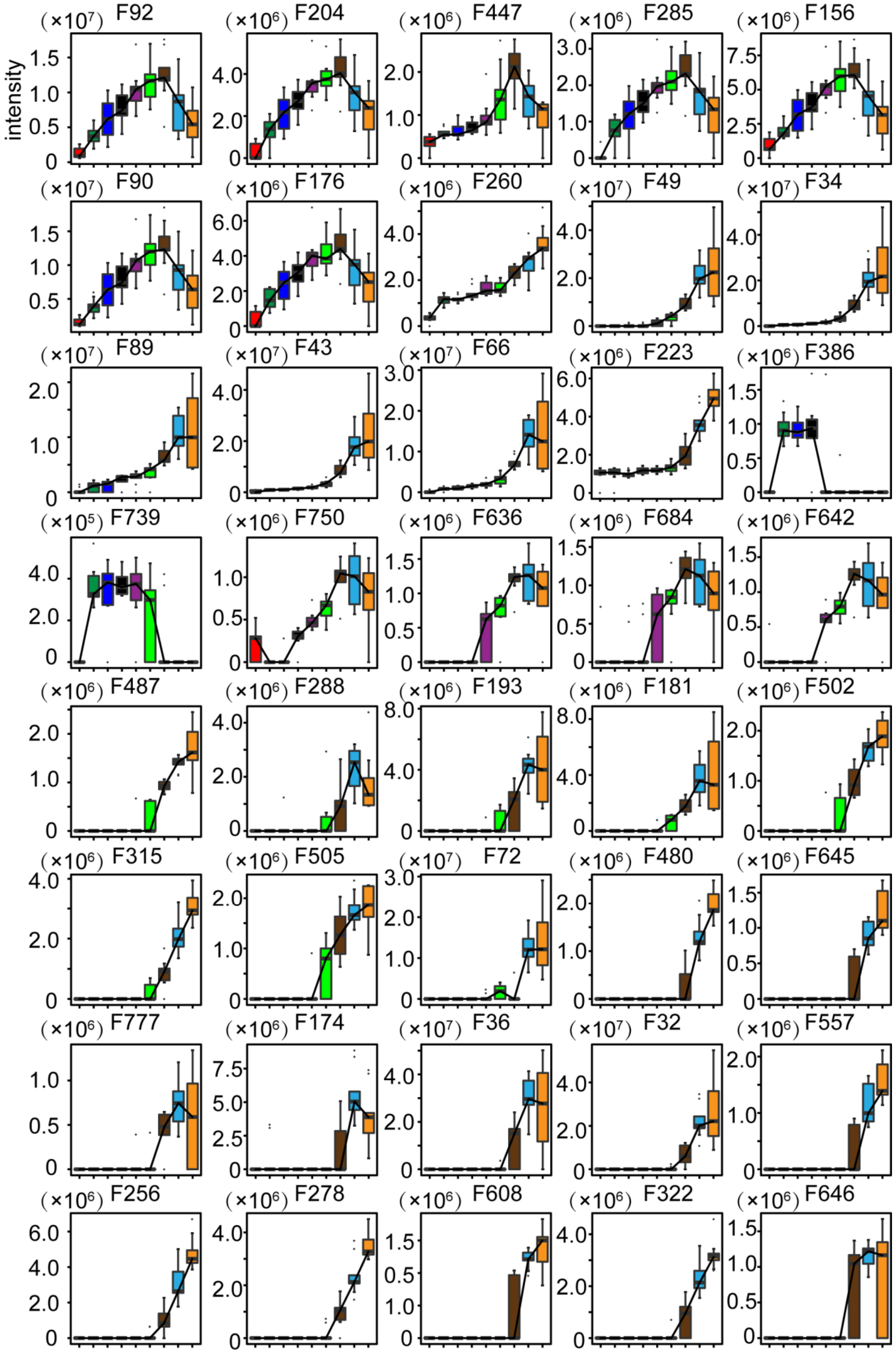
intensity
(×10^7) F92
(×10^6) F204
(×10^6) F447
(×10^6) F285
(×10^6) F156
(×10^7) F90
(×10^6) F176
(×10^6) F260
(×10^7) F49
(×10^7) F34
(×10^7) F89
(×10^7) F43
(×10^7) F66
(×10^6) F223
(×10^6) F386
(×10^5) F739
(×10^6) F750
(×10^6) F636
(×10^6) F684
(×10^6) F642
(×10^6) F487
(×10^6) F288
(×10^6) F193
(×10^6) F181
(×10^6) F502
(×10^6) F315
(×10^6) F505
(×10^7) F72
(×10^6) F480
(×10^6) F645
(×10^6) F777
(×10^6) F174
(×10^7) F36
(×10^7) F32
(×10^6) F557
(×10^6) F256
(×10^6) F278
(×10^6) F608
(×10^6) F322
(×10^6) F646

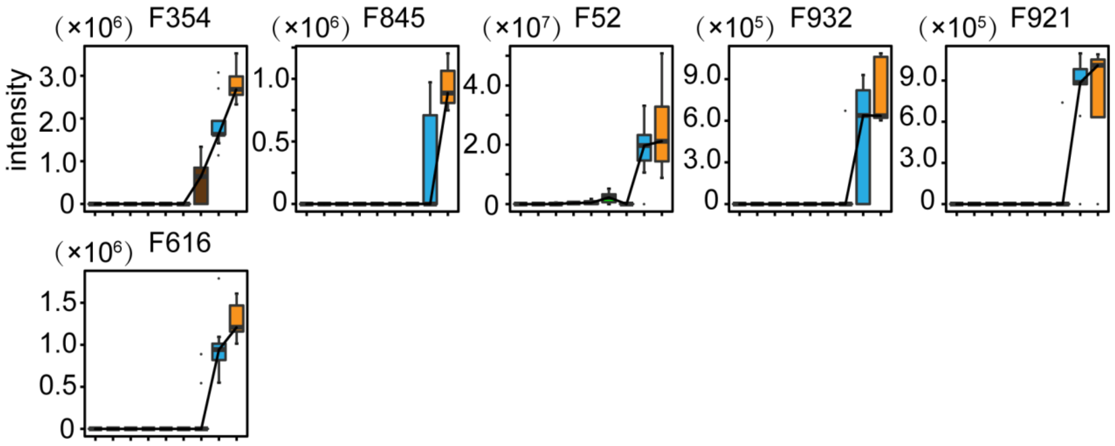

Figure S3. Boxplot of intensities of unidentified differential features at different steaming time, 1 hour (S01, green), 2 hours (S02, blue), 4 hours (S04, black), 8 hours (S08, purple), 12 hours (S12, light green), 24 hours (S24, brown), 48 hours (S48, light blue), 72 hours (S72, yellow), compared with the vacuum freeze dried samples (FD, red).

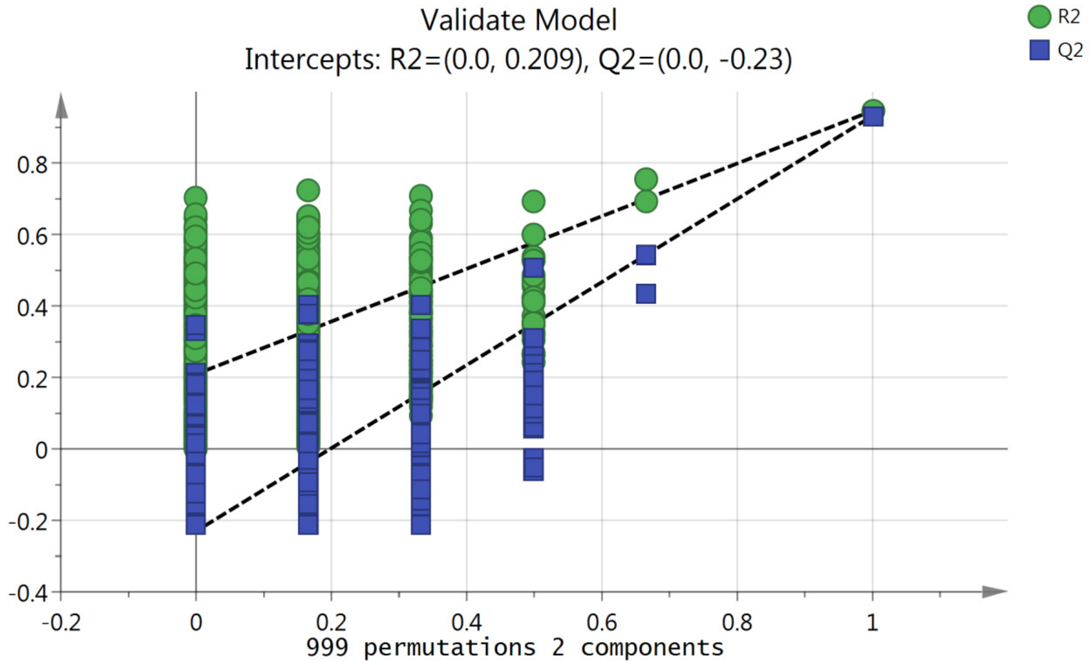

Figure S4. The permutation plot for the PLS model.

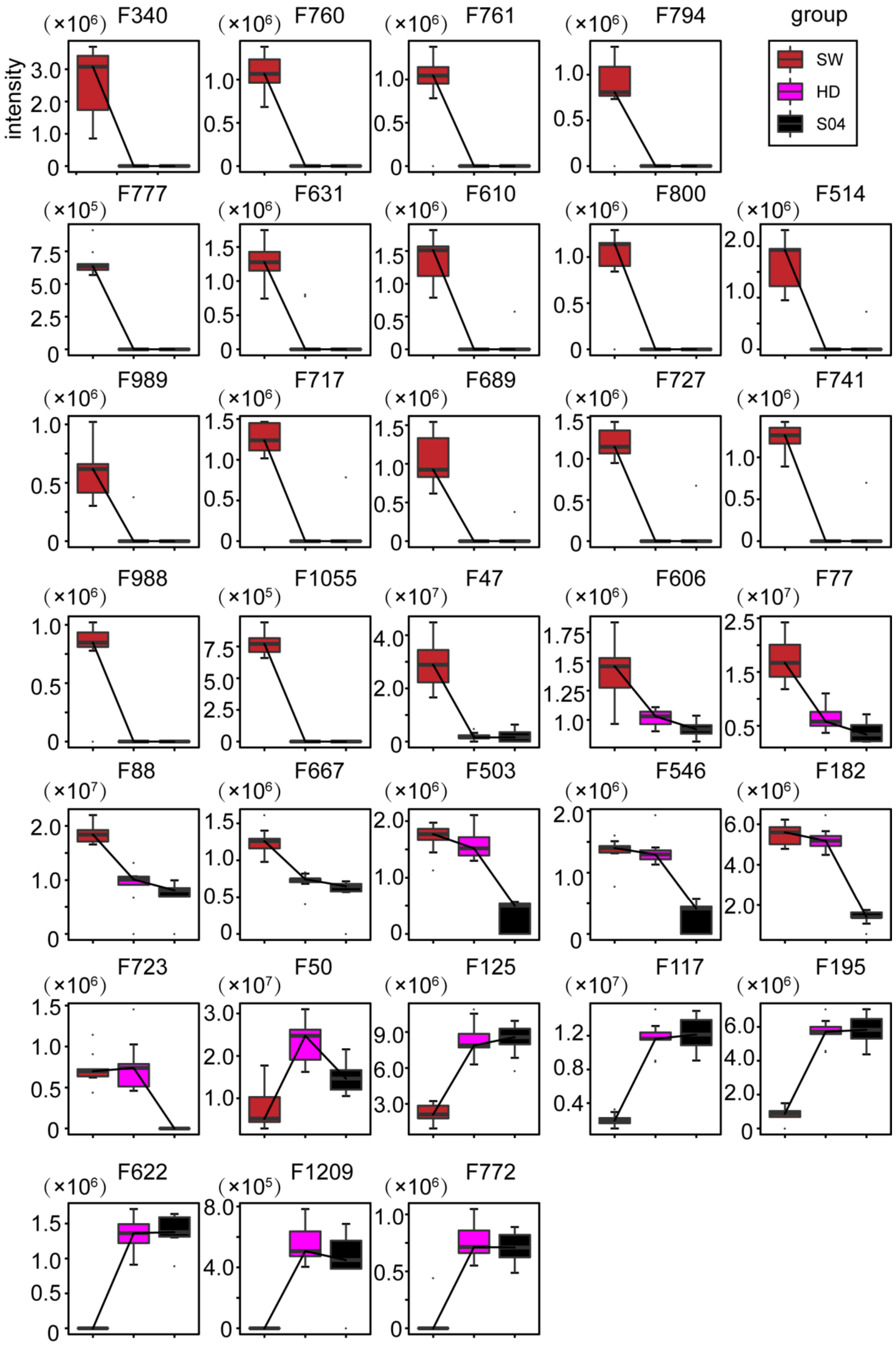

Figure S5. Boxplot of 32 unidentified differential variables in Scrophulariae Radix processed by different methods, sweating (SW, claret), hot-air drying (HD, peach) and steaming for 4 hours (S04, black).
